# The Antidiabetic Drug Metformin Inhibits the Proliferation of Bladder Cancer Cells *in Vitro* and *in Vivo*

**DOI:** 10.3390/ijms141224603

**Published:** 2013-12-18

**Authors:** Tao Zhang, Peng Guo, Yinan Zhang, Hui Xiong, Xiao Yu, Shan Xu, Xinyang Wang, Dalin He, Xunbo Jin

**Affiliations:** 1 Minimally Invasive Urology Center, Provincial Hospital Affiliated to Shandong University, Jinan 250021, China; E-Mails: chris2111@163.com (T.Z.); zhang.yn.2008@gmail.com (Y.Z.); xhui77@163.com (H.X.); surgeonyuxiao@126.com (X.Y.); 2 Department of Urology, the First Hospital of Xi’an Jiaotong University, Xi’an 710061, China; E-Mails: guopeng661@mail.xjtu.edu.cn (P.G.); shanhuxs@163.com (S.X.); wangxinyang0929@163.com (X.W.); 3 Oncology Research Laboratory, Key Laboratory of Environment and Genes Related to Diseases, Ministry of Education, Xi’an Jiaotong University, Xi’an 710061, China

**Keywords:** metformin, bladder cancer, cyclin D1, AMPK, mTOR

## Abstract

Recent studies suggest that metformin, a widely used antidiabetic agent, may reduce cancer risk and improve prognosis of certain malignancies. However, the mechanisms for the anti-cancer effects of metformin remain uncertain. In this study, we investigated the effects of metformin on human bladder cancer cells and the underlying mechanisms. Metformin significantly inhibited the proliferation and colony formation of 5637 and T24 cells *in vitro*; specifically, metformin induced an apparent cell cycle arrest in G0/G1 phases, accompanied by a strong decrease of cyclin D1, cyclin-dependent kinase 4 (CDK4), E2F1 and an increase of p21^waf-1^. Further experiments revealed that metformin activated AMP-activated protein kinase (AMPK) and suppressed mammalian target of rapamycin (mTOR), the central regulator of protein synthesis and cell growth. Moreover, daily treatment of metformin led to a substantial inhibition of tumor growth in a xenograft model with concomitant decrease in the expression of proliferating cell nuclear antigen (PCNA), cyclin D1 and p-mTOR. The *in vitro* and *in vivo* results demonstrate that metformin efficiently suppresses the proliferation of bladder cancer cells and suggest that metformin may be a potential therapeutic agent for the treatment of bladder cancer.

## 1. Introduction

Bladder cancer is the most common malignancy of the urinary tract and one of the major fatal cancers in adult men [1]. Over 70% of the patients are superficial bladder cancer at initial diagnosis, which would be managed with transurethral resection followed by intravesical chemotherapy [2]. However, approximately 60%–70% of these cases will develop recurrent tumors, with 25% showing progression to a higher stage or grade [3]. For advanced bladder cancer, cisplatin-based combination chemotherapy is the current standard therapeutic regimen. Even though bladder cancer is relatively chemotherapy-sensitive, unfortunately, the responses are not typically durable and the majority of these patients experience subsequent disease progression, with a 5-year survival rate of approximately 20%–40% [4,5]. Since the existing treatment options are hardly satisfactory, new effective therapeutic agents with fewer side effects are urgently required for the treatment of bladder cancer.

Metformin (1,1-dimethylbiguanide hydrochloride), an oral biguanide agent, has been widely used for the treatment of type 2 diabetes [6]. Its antihyperglycemic effect mainly relies on the ability to activate the intracellular enzyme AMP-activated protein kinase (AMPK), which leads to reduction of hepatic gluconeogenesis and increase of glucose uptake in peripheral tissues [7,8]. In addition to the antidiabetic properties, it has shown that metformin inhibits the growth of various cancer cells *in vitro* and *in vivo*, including breast cancer [9,10], ovarian cancer [11,12], prostate cancer [13] and gastric cancer [14]. Epidemiological studies also revealed that metformin indeed decreased the incidence of cancer and cancer-related mortality in diabetic patients compared with those treated with other antidiabetic agents [15,16]. Interestingly, a recent study demonstrated that metformin could exert a protective effect on disease recurrence in patients with non-muscle invasive bladder cancer [17], indicating that metformin may be a potential candidate for the development of novel therapeutic agents for bladder cancer. However, the precise mechanism for the anti-cancer effects of metformin on bladder cancer is not entirely elucidated, which needs to be investigated in bladder cancer cell lines and animal models at preclinical level.

In the present study, we investigated the effects of metformin on the proliferation of human bladder cancer cells through cell viability MTT assay, cell count assay and colony formation assay. Then we detected the function of metformin on cell cycle progression by flow cytometry analysis, and further determined whether metformin was involved in AMPK activation and mammalian target of rapamycin (mTOR) inhibition through western blot analysis. More importantly, we also identified whether daily treatment of metformin decreased tumor growth in a xenograft model. Finally, we found that metformin was an efficient inhibitor on the growth of bladder cancer cells.

## 2. Results

### 2.1. Metformin Inhibits the Proliferation of Human Bladder Cancer Cells

**Figure 1.**
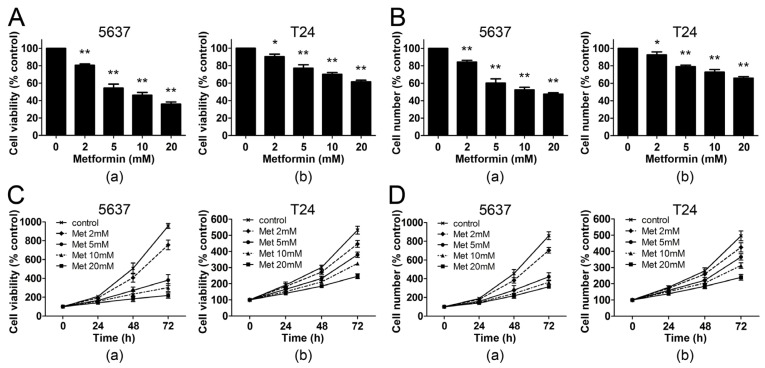
Metformin inhibits the proliferation of bladder cancer cells. (**A**) 5637 (**a**) and T24 (**b**) cells (5 × 10^3^ cells/well) were seeded in 96-well culture plates. After 24 h, cells were treated with metformin (0, 2, 5, 10, 20 mM) for another 48 h. Cell viability was measured by MTT assay. The results were expressed as percent of cell viability compared with control (0 mM). Columns, means of three independent experiments; bars, SEs; (**B**) 5637 (**a**) and T24 (**b**) cells (5 × 10^4^ cells/well) were seeded in 12-well culture plates. After treatment as in panel A, cell numbers were determined using a hemocytometer. The results were expressed as percent of viable cells compared with control. Columns, means of three independent experiments; bars, SEs; (**C**,**D**) 5637 (**a**) and T24 (**b**) cells were treated with metformin (Met) at different concentrations for 24, 48 and 72 h. Cell proliferation was measured by MTT (**C**) or cell count assay (**D**). Data, means of three independent experiments; bars, SEs. *p < 0.05 *versus* control; **p < 0.01 *versus* control.

In order to determine whether metformin affected the proliferation of human bladder cancer cells *in vitro*, we analyzed the effects of the drug on two bladder cancer cell lines: 5637 and T24. Each cell line was grown in 10% FBS and treated with metformin at different concentrations for 48 h. Cell viability was then examined by MTT and cell count assay. As shown in Figure 1A, the MTT viability assay demonstrated that metformin led to a dose-dependent inhibition of cell proliferation in both bladder cancer cell lines. At the concentration of 5 mM, metformin decreased the cell viability of 5637 and T24 cells by 46% and 23% respectively. The results were confirmed with the cell count assay, demonstrating that 5 mM metformin reduced the viable cell number of 5637 and T24 cells by 39% and 21% respectively (Figure 1B). To discern the direct relationship between the decrease in cell viability and the inhibition of cell proliferation, we followed the course of proliferation over three days after the addition of metformin. Both MTT and cell count assay showed that metformin decreased cell proliferation in a dose- and time-dependent manner in 5637 and T24 cells (Figure 1C,D). These results demonstrate that metformin inhibits the proliferation of bladder cancer cells.

### 2.2. Metformin Reduces Colony Formation of Bladder Cancer Cells *in Vitro*

**Figure 2.**
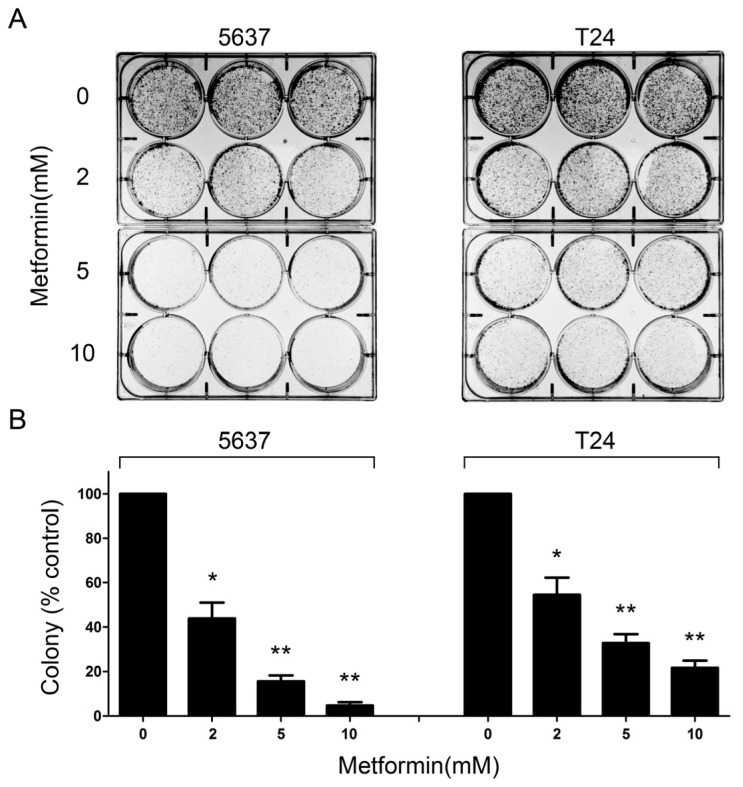
Metformin inhibits colony formation of bladder cancer cells. (**A**) 5637 and T24 cells grown in 6-well culture plates were treated with the indicated concentrations of metformin, every third days for two weeks. The pictures of 6-well culture plates with colonies were taken by a digital camera on day 14; and (**B**) The bar graph was obtained by calculating the percentages of colony numbers relative to controls, defined as 100%, measured by 1-D gel quantity software Quantity One. Columns, means of three independent experiments; bars, SEs. *p < 0.05 *versus* control; **p < 0.01 *versus* control.

To determine whether metformin inhibited anchorage-dependent growth, we examined the colony formation capacity of bladder cancer cells in the presence or absence of metformin for two weeks. We found that metformin reduced the colony formation of 5637 and T24 cells in a dose-dependent manner. As shown in Figure 2A, metformin attenuated clonogenic survival of bladder cancer cells at concentrations as low as 2 mM. At the highest concentration of metformin (10 mM), the colony formation of 5637 and T24 cells was reduced by 95% and 78%, respectively (Figure 2B). These results suggest that metformin treatment could inhibit anchorage-dependent colony formation of bladder cancer cells.

### 2.3. Metformin Induces Cell Cycle Arrest in G0/G1 Phases

**Figure 3.**
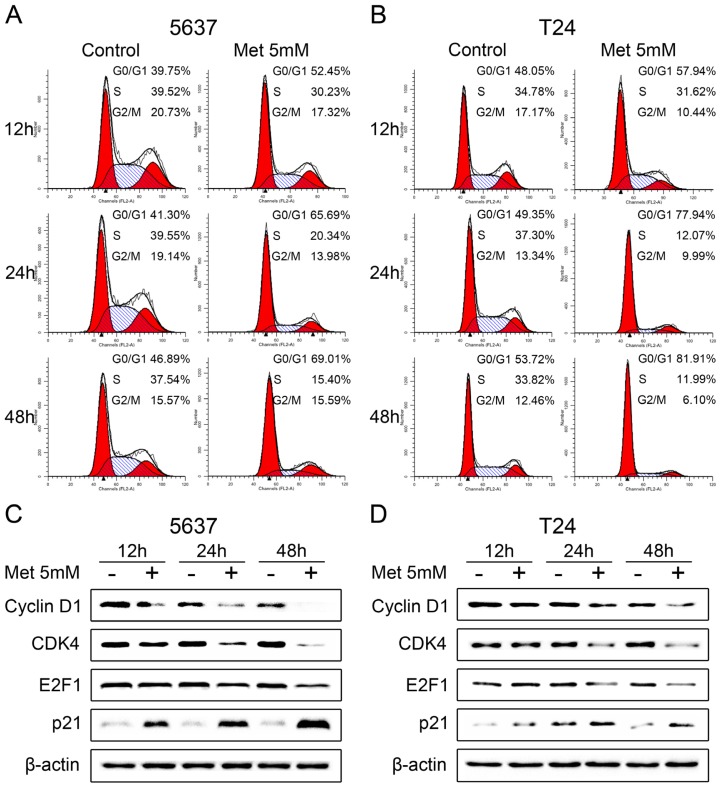
Metformin blocks the cell cycle in G0/G1 phases and affects the expression level of the cell cycle proteins. (**A**,**B**) Proliferating 5637 (**A**) and T24 cells (**B**) were treated with 5 mM metformin (Met) for the indicated time and cell cycle distributions were analyzed by flow cytometry; and (**C**,**D**) Western blot analysis of related cell cycle proteins in 5637 (**C**) and T24 cells (**D**) treated or not with 5 mM metformin (Met) for the indicated time. Data shown are representative of three independent experiments with similar results.

We further investigated the effects of metformin on cell cycle progression by flow cytometry. Proliferating 5637 and T24 cells were treated with 5 mM metformin for 12, 24 and 48 h. As shown in Figure 3A,B, a significantly increase was observed in cells arrested in the G0/G1 phases in both cell lines. In parallel, there was a reduction in the percentage of cells in the S and G2/M phases. To confirm these data, western blot analysis was used to examine the expression of various key proteins implicated in the transition of the G0/G1 phases in 5637 cells with and without metformin treatment. After the addition of 5 mM metformin, the cyclin D1 protein level declined at 12 h and was no longer detectable after 48 h. Concurrently, CDK4 and E2F1 protein levels decreased progressively in response to metformin, while the cyclin-dependent kinase inhibitor p21^waf-1^ increased significantly with metformin treatment in 5637 cells (Figure 3C). Similar results were also obtained in T24 cells (Figure 3D). Taken together, these results demonstrate that metformin affects the expression of key proteins of the cell cycle and induces G0/G1 cell cycle arrest in bladder cancer cells.

### 2.4. Metformin Activates AMPK and Inhibits mTOR Signaling in Bladder Cancer Cells

**Figure 4.**
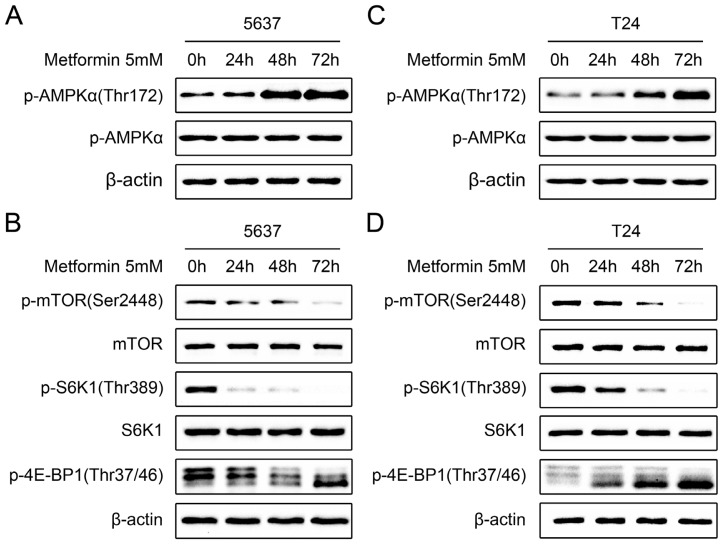
Metformin activates AMP-activated protein kinase (AMPK) and inhibits mammalian target of rapamycin (mTOR) signaling in bladder cancer cells. 5637 and T24 cells were treated with 5 mM metformin for the indicated time and the lysates were immunoblotted with the indicated antibodies. (**A**,**C**) Effects of metformin on the phosphorylation of AMPK in 5637 (**A**) and T24 cells (**C**); and (**B**,**D**) Effects of metformin on mTOR signaling in 5637 (**B**) and T24 cells (**D**). Data shown are representative of three independent experiments.

Metformin has been shown to inhibit proliferation in some cancer cell lines through activation of the AMPK pathway [9]. Therefore, we investigated if the same pathway was involved in the antiproliferative effects of metformin on bladder cancer cells. 5637 cells were treated with 5 mM metformin for 24, 48 and 72 h. Western blot analysis revealed that metformin treatment significantly increased AMPK phosphorylation on Thr172 in a time-dependent manner, indicating that metformin induced AMPK activation in 5637 cells (Figure 4A). Since activation of AMPK has been shown to inhibit the mTOR pathway, a critical translational pathway for protein synthesis [18], we further examined the effects of metformin on mTOR signaling. As shown in Figure 4B, 5 mM metformin treatment resulted in inhibition of mTOR, as demonstrated by time-dependently decreased phosphorylation of mTOR (Ser2448), S6K1 (Thr389), and 4E-BP1 (Thr37/46) in treated 5637 cells, compared with untreated cells. Similar results were also obtained in T24 cells (Figure 4C,D). Collectively, these results suggest that metformin treatment leads to activation of AMPK and inhibition of the mTOR signaling pathway in bladder cancer cells.

### 2.5. Metformin Inhibits the Growth of Human Bladder Tumor Xenografts in Nude Mice

**Figure 5.**
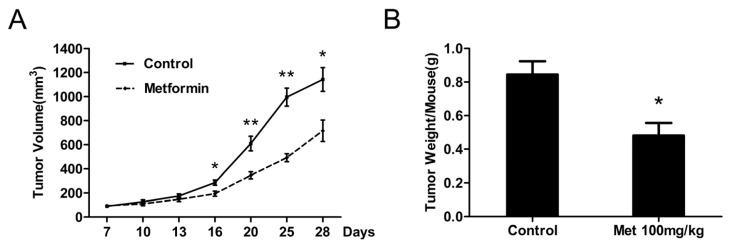

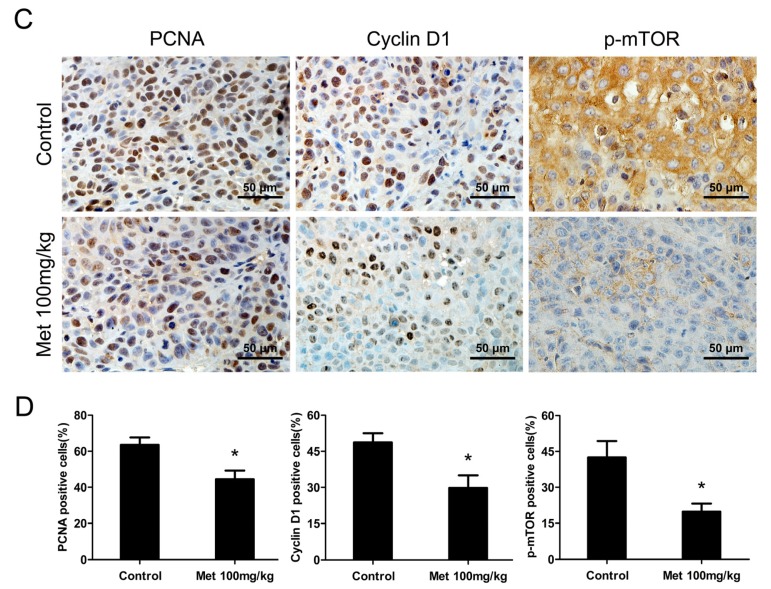
*In vivo* antitumor effects of metformin on bladder cancer xenograft model. Xenografts were generated by implantation of 2 × 10^6^ cells of 5637 cells subcutaneously into the right flanks of nude mice. When the tumors reached a mean diameter of 6 mm, the animals were randomized into control and treated groups (five mice per group). Metformin (Met, 100 mg/kg) was injected once daily intraperitoneally for three weeks, control mice received purified water only. (**A**) Graphs represent the average tumor volumes of 5637 xenografts in mice from the control and metformin-treated groups; (**B**) Weight of the excised tumors from the two groups; (**C**) Representative microphotograph of PCNA, cyclin D1 and p-mTOR staining on tumor sections from the control and metformin-treated groups. The scale bars equal 50 μm; and (**D**) Quantitative data for percentage of PCNA, cyclin D1 and p-mTOR positive cells in tumors from the two groups. Data, means of five mice in each group; bars, SEs. *p < 0.05 *versus* control; **p < 0.01 *versus* control.

Given our results showing the inhibitory effects of metformin on cell signaling and cell proliferation *in vitro*, we examined whether or not this antidiabetic agent could affect tumor growth *in vivo*. Each mouse was inoculated with 5637 cells subcutaneously and metformin was injected daily intraperitoneally at 100 mg/kg body weight per day for three weeks. All mice survived at the end of the experiment and metformin did not affect the animal weight and diet consumption (data not shown). As shown in Figure 5A, intraperitoneal administration of metformin significantly decreased the growth of 5637 tumor xenografts in nude mice. Correspondingly, the average tumor weight in metformin-treated mice was also decreased by 43% (*p* < 0.05) compared with that in the control mice (Figure 5B). In addition, the expression of PCNA, cyclin D1 and p-mTOR in xenograft tumors were evaluated by immunohistochemistry. Lesser number of PCNA immunoreactivity was observed in metformin-treated mice, which accounted for 19% decrease (*p* < 0.05) in PCNA-positive cells compared with control (Figure 5C,D, left panel), demonstrating significant inhibitory effects of metformin on tumor cell proliferation *in vivo*. Furthermore, metformin treatment also markedly reduced the expression of cyclin D1 and p-mTOR, which was confirmed by the scoring of percentage positive cells under 400× magnification in ten randomly selected areas in each tumor sample (Figure 5C,D, middle and right panel). These data are consistent with our *in vitro* results and demonstrate, for the first time, that metformin modulates cell cycle and protein synthesis to restrain the growth of human bladder tumor xenografts in nude mice.

## 3. Discussion

Metformin is an oral antidiabetic agent used for the treatment of type 2 diabetes and has the clinical advantage of being highly effective with minimal toxicity. Recent studies indicated that metformin reduced the risk of cancer and inhibited the proliferation of various cancer cells *in vitro* and *in vivo*, such as breast cancer [9,10], ovarian cancer [11,12], prostate cancer [13] and gastric cancer [14]. However, the anti-cancer effects of metformin on bladder cancer remain unknown. Here, we demonstrate for the first time that metformin not only effectively inhibits the proliferation and clonogenicity of human bladder cancer cells *in vitro*, but also suppresses the tumor growth in a xenograft model when administered intraperitoneally.

Downregulation of cyclin D1 in response to metformin has been shown in several cancer cell lines. In prostate cancer cells, metformin treatment led to a dose-dependent inhibition of proliferation through a decrease in cyclin D1 levels, correlated with blocking cell cycle in the G0/G1 phases [13]. Downregulation of cell cycle proteins, including cyclin D1, CDK4 and CDK6, was also observed in metformin-treated gastric cancer cells [14]. Our results also demonstrated that metformin could arrest bladder cancer cells in the G0/G1 phases with concomitant decreases in the expression of cyclin D1, CDK4 and E2F1. Cyclin D1 plays a pivotal role in the regulation of cell cycle transitions in mammalian cells, it binds to CDK4 and CDK6 to form active cyclin/CDK complexes that help to phosphorylate the retinoblastoma protein (Rb). Then phosphorylation Rb releases the transcription factor E2F, which activates the transcription of genes required for G1/S phases transition [19]. Thus downregulation of cyclin D1 may be essential for the anti-cancer effects of metformin in bladder cancer cells. Previous reports have shown that the cyclin D1 gene (CCND1) is amplified and/or overexpressed in several human tumors including bladder cancer [20,21]. Overexpression of cyclin D1 appeared to play an important role in the early stage of urothelial tumorigenesis and correlated with early recurrence in superficial bladder cancers [22]. Moreover, the polymorphism within the cyclin D1 gene was associated with risk and clinicopathologic characteristics of urinary bladder cancer [23,24]. Therefore, inhibition of cyclin D1 seems to be an effective molecular target for the treatment of bladder cancer. Our *in vivo* study also showed that metformin could decrease the expression levels of cyclin D1 in a bladder cancer xenograft model and suggests that metformin may be a valuable potential therapeutic agent to block bladder tumor growth.

In the present study, metformin activated the AMPK pathway in human bladder cancer cells as seen in other cell types [9]. AMPK is a serine/threonine kinase that acts as a cellular energy sensor maintaining the energy balance in the eukaryotic cells [25]. It is activated in response to cellular stresses that deplete cellular energy levels and increase the AMP/ATP ratio [26,27]. The antihyperglycemic effect of metformin mainly relies on its ability to activate AMPK, leading to inhibition of gluconeogenesis in liver and increase of glucose uptake in peripheral tissues [7,8]. In addition to the metabolic effects, activation of AMPK has been recognized as an attractive anti-cancer therapeutic strategy [28]. Some researches demonstrated that the antiproliferative action of metformin was exactly via activation of AMPK and small interfering RNAs against AMPK (α1 subunit) or AMPK inhibitors could rescue cells from metformin-induced growth inhibition [9,29]. Activation of AMPK has been shown to inhibit its downstream target, mTOR, which plays a central role in cell growth and proliferation [18]. It is the AMPK-mediated mTOR inhibition that is supposed to be the crucial factor responsible for the antitumor properties of metformin [30]. Our study also demonstrated that mTOR signaling pathway was inhibited by metformin in bladder cancer cells, as evidenced by the decreased phosphorylation of mTOR, S6K1, and 4E-BP1. These data indicate that metformin activates AMPK in bladder cancer cells, leading to inhibition of mTOR signaling pathway and thus a reduced cellular proliferation. Previous studies suggested that mTOR was activated in most bladder caners and increased p-mTOR status was associated with worsened pathological stage and shortened patient survival [31]. Moreover, inhibition of mTOR signaling pathway in bladder cancer models demonstrated remarkable anti-cancer activity both *in vitro* and *in vivo* [32–34], making it an attractive target for cancer therapeutics. Taken together, our study reveals that metformin may be a potential therapeutic agent to treat bladder cancer. On the other hand, a study of Sahra *et al.* showed that metformin could still inhibit mTOR pathway in prostate cancer cells even in the absence of AMPK activation [13]. Other groups also observed that metformin could hinder the proliferation of AMPK null mouse embryo fibroblasts and AMPK silenced ovarian cancer cells [11]. This disparity may be due to a cell specific effect and need further clarification. The tumor suppressor liver kinase B1 (LKB1) has been identified as the key upstream serine/threonine kinase that activates AMPK [28]. Recent studies demonstrated that cancer cells lacking LKB1 protein expression do not respond to metformin *in vitro*, indicating LKB1 may play an important role in mediating the anti-cancer effects of metformin [11,35]. In bladder cancers, LKB1 was not a critical mutation target unlike other sporadic cancers and both 5637 and T24 were LKB1 wild-type cancer cell lines [36,37]. Future studies are required to investigate the role of LKB1 in the metformin-mediated anti-cancer effects in bladder cancer cells.

Our *in vitro* study was conducted using higher doses of metformin in millimolar range, from 2 to 20 mM, which were coincident with those of similar pre-clinical and *in vitro* studies in other cancer cell types [9,11]. But the use of such higher doses has been the subject of criticism since it seems unattainable *in vivo*. Indeed, the plasma concentration of metformin in type 2 diabetic patients treated with the drug approximates 50 μM [38], thus the dose range employed in the present study is 100-fold higher than the therapeutic levels. However, it must be noted that the high concentrations of glucose and FBS in culture medium are excessive stimulators for cell growth and it may need a higher dose to see the anti-cancer effects of metformin in cell culture systems. Moreover, it has been reported that metformin accumulates in tissues at much higher concentrations than in the blood [39,40], indicating that the concentrations of metformin employed *in vitro* study (1–10 mM) might be attained during cancer treatment. Therefore, the present results still seem clinically relevant. Furthermore, our *in vivo* study showed that metformin markedly inhibited the growth of human bladder tumor in a xenograft model. The concentration of metformin administered to the animal was 100 mg/kg body weight per day, which was equivalent to 480 mg/60 kg per day in humans. Given that the maximum recommended daily dose of metformin for the treatment of type 2 diabetic patients is 2550 mg per day, our study indicates that metformin could exert its anti-cancer ability *in vivo* even at a safe dose level. Collectively, the remarkable efficiency of metformin to inhibit bladder cancer cells proliferation and tumor growth may have important implications in the treatment of bladder cancer.

## 4. Experimental Section

### 4.1. Reagents

Metformin (1,1-dimethylbiguanide hydrochloride) was purchased from Sigma-Aldrich (St. Louis, MO, USA) and diluted across a range of concentrations at 2, 5, 10 and 20 mM in culture media. Antibodies against AMPKα, phospho-AMPKα (Thr172), mTOR, phospho-mTOR (Ser2448), S6 Kinase 1 (S6K1), phospho-S6K1 (Thr389), phospho-eukaryotic translation initiation factor 4E (eIF4E)-binding protein 1 (p-4E-BP1) (Thr37/46), peroxidase-conjugated secondary antibodies were obtained from Cell Signaling Technology (Danvers, MA, USA). Antibodies against cyclin D1, cyclin-dependent kinase 4 (CDK4), E2F1, P21, proliferating cell nuclear antigen (PCNA) and β-actin were from Santa Cruz Biotechnology (Santa Cruz, CA, USA). All other reagents were purchased from Sigma-Aldrich (St. Louis, MO, USA) unless otherwise specified.

### 4.2. Cell Lines and Culture Conditions

The human bladder transitional cell carcinoma cell line 5637 and T24 were from the American Type Culture Collection (ATCC, Rockville, MD, USA) and cultured in fewer than 6 months after resuscitation. Cells were cultured in RPMI-1640 (5637) or Dulbecco’s Modified Eagle’s Medium (DMEM; T24) supplemented with 10% of fetal bovine serum (FBS) and 1% of penicillin-streptomycin at 37 °C, in humidified air containing 5% of CO_2_. Cell culture media and FBS were obtained from Invitrogen (Carlsbad, CA, USA). Cells were passaged with 0.25% trypsin-EDTA when they reached ~80% confluence.

### 4.3. Cell Viability Assay

Cell viablility was assessed using a tetrazolium-based assay. Briefly, cells were seeded at 5 × 10^3^ per well in 96-well culture plates and incubated in medium containing 10% FBS. After 24 h, cells were treated with metformin (0, 2, 5, 10, 20 mM) for 24, 48 and 72 h. At the indicated time points, cells were washed once and incubated with 0.5 mg/mL of 3-(4,5-dimethylthiazol-2-yl)-2,5-diphenyl tetrazolium bromide (MTT) at 37 °C for 4 h. Then the medium was discarded carefully and dimethyl sulfoxide (DMSO) was added to solubilize the formazan crystals. Finally, the absorbance was measured for each well at a wavelength of 490 nm using the Microplate Autoreader (Bio-Tek Instruments Inc., Winooski, VT, USA). Independent experiments were repeated in triplicate.

### 4.4. Cell Count Assay

Cells were seeded at 5 × 10^4^ per well in 12-well culture plates. After 24 h, cells were incubated in the absence or presence of metformin for 24, 48 and 72 h. At the indicated time points, cells were washed twice with phosphate buffered saline (PBS) to remove any loosely attached or floating cells. The cells were then harvested and immediately stained with 0.2% trypan blue. Viable cells were quantified using a hemocytometer under an inverted microscope. Independent experiments were repeated in triplicate.

### 4.5. Colony Formation Assay

Cells were seeded in 6-well culture plates in triplicates at a density of 1000 cells/well in 2 mL medium containing 10% FBS. After 24 h, cultures were replaced with fresh medium containing 5% FBS as control, or the same medium containing 2, 5 or 10 mM metformin. The plates were incubated at 37 °C with 5% CO_2_ in a humidified incubator for 14 days and media were replaced every third day. Then the colonies were fixed with 4% paraformaldehyde, stained in 0.5% crystal violet solution for 15 min at room temperature, and washed with distilled water to remove excess dye. The colony numbers were obtained by 1-D gel analysis software Quantity One (Bio-Rad Laboratories Inc., Hercules, CA, USA).

### 4.6. Flow Cytometric Analysis

Flow cytometric analysis was performed to evaluate the effects of metformin on cell cycle distribution. In brief, cells grown in 6-well culture plates (2.0 × 10^5^ cells/well) were treated with 5 mM metformin or without metformin for 12, 24 and 48 h. After treatments, cells were harvested by trypsinization, washed twice with ice-cold PBS, and then fixed overnight at −20 °C in 70% ethanol. Before analysis, cells were washed with PBS and incubated with 50 μg/mL propidium iodide (PI) and 50 μg/mL RNase A in PBS at room temperature for 30 min. Then flow cytometry was performed using a FACSCalibur (Becton Dickinson, San Jose, CA, USA) system with CELLQuest software (Version 3.3; Becton Dickinson, San Jose, CA, USA) and the cell cycle distributions were calculated by ModFit LT software (Version 3.0; Verity Software House, Topsham, ME, USA). All experiments were carried out in triplicate to assess the consistency of response.

### 4.7. Protein Extraction and Western Blot Analysis

After treatment for the indicated time, cells were washed thrice with ice-cold PBS and lysed in lysis buffer (10 mM of Tris-HCl (pH of 7.4), 150 mM of NaCl, 0.1% of SDS, 1 mM of EDTA, 1 mM of EGTA, 0.3 mM of PMSF, 0.2 mM of sodium orthovanadate, 1% of NP-40, 10 mg/mL of leupeptin and 10 mg/mL of aprotinin) on ice for 30 min. The lysates were then centrifuged at 15,000 rpm for 15 min at 4 °C and the supernatants were collected for protein concentration determination. For immunoblot analyses, equal amounts of protein (30–60 μg) were subjected to SDS-PAGE on 8% or 12% Tris-glycine gels, and separated proteins were transferred onto nitrocellulose membranes by western blot. The membranes were blocked with 5% skim milk in Tris Buffered Saline (TBS) for 1 h at room temperature and probed with primary antibodies against desired molecules overnight at 4 °C. After washing in TBS-Tween (0.1%), the membranes were incubated with horseradish peroxidase-conjugated secondary antibody for 1 h at room temperature. Protein signal was then detected with the ECL chemiluminescent detection system (Bio-Rad, Hercules, CA, USA). β-actin was used as a loading control.

### 4.8. Tumor Xenograft Model

Four-week-old male athymic BALB/c nu/nu mice were obtained from the Experimental Animal Center of Xi’an Jiaotong University (Xi’an, China). Animal care and protocols were approved by the Institutional Animal Care and Use Committee of Xi’an Jiaotong University and the permit number was SCXK2012-2096. The animal experiments were performed in adherence with the NIH Guidelines on the Use of Laboratory Animals [41]. To establish 5637 tumors in mice, the early passage 5637 cells were harvested and resuspended in serum-free RPMI-1640 medium. Cells (2 × 10^6^ cells in 100 μL) were then injected subcutaneously into the right flank regions of each mouse to initiate tumor growth. After 7 days, the tumors reached a mean diameter of 6 mm in all recipients. Then tumor-bearing mice were randomly divided into control and treated groups (5 mice per group). The metformin-treated group was injected intraperitoneally at 100 mg/kg body weight per day for 3 weeks and the control group received vehicle only. Body weight and diet consumption were recorded twice weekly throughout the study. After the initiation of the metformin administration, the tumor size was measured with an external caliper twice a week and tumor volume was calculated by the formula 0.5236 × A × B^2^, wherein A represents the long axis and B represents the short axis of the tumor. At the end of experiment, all the animals were sacrificed and the tumors were excised, weighed, then fixed in 4% paraformaldehyde and embedded in paraffin for further analyses.

### 4.9. Immunohistochemistry

Paraffin-embedded tissue sections (5-μm thick) were processed and immunohistochemistry (IHC) was performed using a Dako Autostainer Plus system (DakoCytomation, Carpinteria, CA, USA). In brief, sections were deparaffinized, rehydrated, subjected to 5 min of heat-induced antigen retrieval, 15 min of endogenous enzyme block, and incubated with primary antibody at 4 °C overnight. Next day, after 30 min of DakoCytomation EnVision-HRP reagent (DakoCytomation, Carpinteria, CA, USA) incubation at room temperature, the signals were detected by adding substrate hydrogen peroxide using diaminobenzidine (DAB) as a chromogen followed by hematoxylin counterstaining. Negative controls were incubated only with primary antibody dilution buffer under identical conditions. All sections were examined under a light microscope and positive stained cells (brown) were quantified as number of positive cell × 100/total number of cells in 10 random microscopic (400×) fields in each slice.

### 4.10. Statistical Analysis

Data are presented as the mean ± SE from three independent experiments, and statistical analysis was done using a Student’s *t* test by software of SPSS 15.0 (SPSS Inc., Chicago, IL, USA). The value of *p* < 0.05 was considered statistically significant.

## 5. Conclusions

In summary, our study demonstrates that metformin effectively inhibits human bladder cancer cell proliferation and tumor growth *in vitro* and *in vivo*, possibly by inducing G0/G1 cell cycle arrest and activating AMPK/mTOR pathway. These results suggest that metformin could be a potential candidate for the development of novel treatment strategies for human bladder cancer.

## References

[1] Siegel R., Ward E., Brawley O., Jemal A. (2011). Cancer statistics, 2011: The impact of eliminating socioeconomic and racial disparities on premature cancer deaths. CA Cancer J. Clin.

[2] Bischoff C.J., Clark P.E. (2009). Bladder cancer. Curr. Opin. Oncol.

[3] Schenk-Braat E.A., Bangma C.H. (2005). Immunotherapy for superficial bladder cancer. Cancer Immunol. Immunother.

[4] Herr H.W., Dotan Z., Donat S.M., Bajorin D.F. (2007). Defining optimal therapy for muscle invasive bladder cancer. J. Urol.

[5] Choueiri T.K., Raghavan D. (2008). Chemotherapy for muscle-invasive bladder cancer treated with definitive radiotherapy: Persisting uncertainties. Nat. Clin. Pract. Oncol.

[6] Witters L.A. (2001). The blooming of the French lilac. J. Clin. Invest.

[7] Zhou G., Myers R., Li Y., Chen Y., Shen X., Fenyk-Melody J., Wu M., Ventre J., Doebber T., Fujii N. (2001). Role of AMP-activated protein kinase in mechanism of metformin action. J. Clin. Invest.

[8] Boyle J.G., Salt I.P., McKay G.A. (2010). Metformin action on AMP-activated protein kinase: A translational research approach to understanding a potential new therapeutic target. Diabet. Med.

[9] Zakikhani M., Dowling R., Fantus I.G., Sonenberg N., Pollak M. (2006). Metformin is an AMP kinase-dependent growth inhibitor for breast cancer cells. Cancer Res.

[10] Anisimov V.N., Berstein L.M., Egormin P.A., Piskunova T.S., Popovich I.G., Zabezhinski M.A., Kovalenko I.G., Poroshina T.E., Semenchenko A.V., Provinciali M. (2005). Effect of metformin on life span and on the development of spontaneous mammary tumors in HER-2/neu transgenic mice. Exp. Gerontol.

[11] Rattan R., Giri S., Hartmann L.C., Shridhar V. (2011). Metformin attenuates ovarian cancer cell growth in an AMP-kinase dispensable manner. J. Cell. Mol. Med.

[12] Rattan R., Graham R.P., Maguire J.L., Giri S., Shridhar V. (2011). Metformin suppresses ovarian cancer growth and metastasis with enhancement of cisplatin cytotoxicity *in vivo*. Neoplasia.

[13] Ben S.I., Laurent K., Loubat A., Giorgetti-Peraldi S., Colosetti P., Auberger P., Tanti J.F., le Marchand-Brustel Y., Bost F. (2008). The antidiabetic drug metformin exerts an antitumoral effect *in vitro* and *in vivo* through a decrease of cyclin D1 level. Oncogene.

[14] Kato K., Gong J., Iwama H., Kitanaka A., Tani J., Miyoshi H., Nomura K., Mimura S., Kobayashi M., Aritomo Y. (2012). The antidiabetic drug metformin inhibits gastric cancer cell proliferation *in vitro* and *in vivo*. Mol. Cancer Ther.

[15] Noto H., Goto A., Tsujimoto T., Noda M. (2012). Cancer risk in diabetic patients treated with metformin: A systematic review and meta-analysis. PLoS One.

[16] Evans J.M., Donnelly L.A., Emslie-Smith A.M., Alessi D.R., Morris A.D. (2005). Metformin and reduced risk of cancer in diabetic patients. BMJ.

[17] Rieken M., Xylinas E., Kluth L., Crivelli J.J., Chrystal J., Faison T., Lotan Y., Karakiewicz P.I., Fajkovic H., Babjuk M. (2013). Association of diabetes mellitus and metformin use with oncological outcomes of patients with non-muscle invasive bladder cancer. BJU Int.

[18] Kimball S.R. (2006). Interaction between the AMP-activated protein kinase and mTOR signaling pathways. Med. Sci. Sports Exerc.

[19] Sherr C.J. (1996). Cancer cell cycles. Science.

[20] Bringuier P.P., Tamimi Y., Schuuring E., Schalken J. (1996). Expression of cyclin D1 and EMS1 in bladder tumours; relationship with chromosome 11q13 amplification. Oncogene.

[21] Schuuring E., Verhoeven E., Mooi W.J., Michalides R.J. (1992). Identification and cloning of two overexpressed genes, U21B31/PRAD1 and EMS1, within the amplified chromosome 11q13 region in human carcinomas. Oncogene.

[22] Shin K.Y., Kong G., Kim W.S., Lee T.Y., Woo Y.N., Lee J.D. (1997). Overexpression of cyclin D1 correlates with early recurrence in superficial bladder cancers. Br. J. Cancer.

[23] Yuan L., Gu X., Shao J., Wang M., Wang M., Zhu Q., Zhang Z. (2010). Cyclin D1 G870A polymorphism is associated with risk and clinicopathologic characteristics of bladder cancer. DNA Cell Biol.

[24] Lin H.H., Ke H.L., Hsiao K.H., Tsai C.W., Wu W.J., Bau D.T., Chang L.L. (2011). Potential role of CCND1 G870A genotype as a predictor for urothelial carcinoma susceptibility and muscle-invasiveness in Taiwan. Chin. J. Physiol.

[25] Kahn B.B., Alquier T., Carling D., Hardie D.G. (2005). AMP-activated protein kinase: Ancient energy gauge provides clues to modern understanding of metabolism. Cell Metab.

[26] Hardie D.G. (2008). AMPK: A key regulator of energy balance in the single cell and the whole organism. Int. J. Obes.

[27] Towler M.C., Hardie D.G. (2007). AMP-activated protein kinase in metabolic control and insulin signaling. Circ. Res.

[28] Luo Z., Zang M., Guo W. (2010). AMPK as a metabolic tumor suppressor: Control of metabolism and cell growth. Future Oncol.

[29] Zakikhani M., Dowling R.J., Sonenberg N., Pollak M.N. (2008). The effects of adiponectin and metformin on prostate and colon neoplasia involve activation of AMP-activated protein kinase. Cancer Prev. Res.

[30] Wysocki P.J., Wierusz-Wysocka B. (2010). Obesity, hyperinsulinemia and breast cancer: Novel targets and a novel role for metformin. Expert. Rev. Mol. Diagn.

[31] Hansel D.E., Platt E., Orloff M., Harwalker J., Sethu S., Hicks J.L., de Marzo A., Steinle R.E., Hsi E.D., Theodorescu D. (2010). Mammalian target of rapamycin (mTOR) regulates cellular proliferation and tumor growth in urothelial carcinoma. Am. J. Pathol.

[32] Mansure J.J., Nassim R., Chevalier S., Rocha J., Scarlata E., Kassouf W. (2009). Inhibition of mammalian target of rapamycin as a therapeutic strategy in the management of bladder cancer. Cancer Biol. Ther.

[33] Seager C.M., Puzio-Kuter A.M., Patel T., Jain S., Cordon-Cardo C., Mc K.J., Abate-Shen C. (2009). Intravesical delivery of rapamycin suppresses tumorigenesis in a mouse model of progressive bladder cancer. Cancer Prev. Res.

[34] Pinto-Leite R., Botelho P., Ribeiro E., Oliveira P.A., Santos L. (2009). Effect of sirolimus on urinary bladder cancer T24 cell line. J. Exp. Clin. Cancer Res.

[35] Morgillo F., Sasso F.C., Della C.C., Vitagliano D., D’Aiuto E., Troiani T., Martinelli E., de Vita F., Orditura M., de Palma R. (2013). Synergistic effects of metformin treatment in combination with gefitinib, a selective EGFR tyrosine kinase inhibitor, in LKB1 wild-type NSCLC cell lines. Clin. Cancer Res.

[36] Platt F.M., Hurst C.D., Taylor C.F., Gregory W.M., Harnden P., Knowles M.A. (2009). Spectrum of phosphatidylinositol 3-kinase pathway gene alterations in bladder cancer. Clin. Cancer Res.

[37] Guo Y., Chekaluk Y., Zhang J., Du J., Gray N.S., Wu C.L., Kwiatkowski D.J. (2013). TSC1 involvement in bladder cancer: Diverse effects and therapeutic implications. J. Pathol.

[38] Martin-Castillo B., Vazquez-Martin A., Oliveras-Ferraros C., Menendez J.A. (2010). Metformin and cancer: doses, mechanisms and the dandelion and hormetic phenomena. Cell Cycle.

[39] Owen M.R., Doran E., Halestrap A.P. (2000). Evidence that metformin exerts its anti-diabetic effects through inhibition of complex 1 of the mitochondrial respiratory chain. Biochem. J.

[40] Wilcock C., Bailey C.J. (1994). Accumulation of metformin by tissues of the normal and diabetic mouse. Xenobiotica.

[41] National Research Council (1996). Guide for the Care and Use of Laboratory Animals.

